# Changes in the gut microbiota of esophageal carcinoma patients based on 16S rRNA gene sequencing: a systematic review and meta-analysis

**DOI:** 10.3389/fonc.2024.1366975

**Published:** 2024-08-29

**Authors:** Li Zhang, Delin Li, Yongsheng Zhang, Wenqi Hu, Haoyue Lv, Xiaodong Zhang, Hongyu Zhang

**Affiliations:** ^1^ Department of Pharmacology, Jinan Central Hospital Affiliated to Shandong First Medical University, Jinan, China; ^2^ Department of Medical Equipment, Jinan Mental Health Center, Jinan, China; ^3^ Department of Health Management, The First Affiliated Hospital of Shandong First Medical University, Jinan, China; ^4^ Postgraduate Department, Shandong First Medical University (Shandong Academy of Medical Sciences), Jinan, China

**Keywords:** esophageal carcinoma, microbiome, intestinal, 16S ribosomal RNA (rRNA) sequencing, meta-analysis

## Abstract

**Background:**

This study conducts a systematic review through meta-analysis, comparing the composition and diversity of the gut microbiome in patients with esophageal cancer and healthy individuals, and explores the relationship between risk factors and related factors of esophageal cancer.

**Methods:**

According to the Preferred Reporting Items for Systematic Reviews and Meta-Analyses (PRISMA), we comprehensively searched the databases of PubMed, Web of Science, Embase, Cochrane Library. In addition, we applied the R programming language version 4.0.3 and Stata 15.1 software for data analysis. We also implemented the Newcastle-Ottawa Scale (NOS), funnel plot analysis, Egger’s test, and Begg’s test to assess the risk of bias.

**Results:**

In this study, a total of 328 studies were identified through the literature search. Among them, 117 duplicate studies were removed, and 202 studies were excluded based on inclusion and exclusion criteria. Finally, 9 studies were included in the analysis, involving a total of 216 patients with esophageal carcinoma and 352 healthy controls. Four studies provided Chao1 index for quantitative consolidation (ES = 637.41, 95% CI: 549.16 to 725.66, p = 0.000, I^2^ = 98.2%). Two studies [27, 29] reported ACE index (ES = 438.89, 95% CI: 362.42 to 515.35, p = 0.000, I^2^ = 97%). Seven studies [26,27,29,30,32] reported the Shannon index for quantitative consolidation (ES = 4.38, 95% CI: 3.95 to 4.81, p = 0.000, I^2^ = 99%). At the phylum level, the abundance of Bacteroidetes(ES = 37.8, 95% CI: 25.75 to 49.85, p = 0.000, I2 = 87.2%) and Proteobacteria(ES = 7.48, 95% CI: 5.02 to 8.85, p = 0.04, I^2^ = 2.4%) have statistical difference between ESCC and HC. There was no significant difference between ESCC and HC in the abundance of genera(p>0.05).

**Conclusions:**

This observational meta-analysis revealed that changes in the GM were correlated with esophageal carcinoma, and variations in some advantageous GM might involve regional differences. Additionally, the study aims to facilitate early diagnosis of esophageal cancer and improve screening and diagnostic efficiency.

## 1 Introduction

Esophageal cancer is a common and highly malignant tumor of the digestive system, and its morbidity and mortality rates continue to increase globally (1). Esophageal cancer ranks eighth among the most commonly diagnosed cancers and sixth among the leading causes of cancer-related deaths worldwide (2). The survival rates for esophageal cancer are still relatively low, with most countries reporting 5-year survival rates of only 10-30% after diagnosis (3). According to the International Agency for Research on Cancer, esophageal cancer causes hundreds of thousands of deaths worldwide each year (2). Based on a stable incidence rate, it is projected that there will be 957,000 new cases of esophageal cancer (including 141,300 cases of adenocarcinoma and 806,000 cases of squamous cell carcinoma) and 880,000 deaths from esophageal cancer in 2040 (4).

The human gut comprises an intricate ecosystem housing billions of microorganisms, which include bacteria, archaea, fungi, protozoa, and viruses, totaling in the trillions (5, 6). The gut microbiome, with its complex array of microorganisms, plays a crucial role in maintaining the body’s immune system, facilitating effective digestion and nutrient absorption, and regulating metabolic processes. In recent research, specific associations have been identified between the composition and diversity of the gut microbiome and certain types of cancer (7, 8). Imbalance of intestinal flora may be associated with the development and progression of esophageal cancer. Research has shown that there are significant differences in the abundance and diversity of gut microbiota between individuals with esophageal cancer and healthy individuals (10, 20, 21). Deng et al. analyzed the intestinal flora of esophageal cancer patients and healthy controls and found that the microbial abundance of the intestinal flora of esophageal cancer patients was higher than that of healthy controls (10). Analyzing the intestinal flora of esophageal cancer patients can help us understand the pathogenesis of esophageal cancer and reveal the interaction between esophageal cancer and intestinal flora. Identification of potential microbial biomarkers is essential for risk assessment, early diagnosis, prognosis and personalized treatment of esophageal cancer, and can improve the efficiency of screening and diagnosis.

The 16S rRNA genes are universally present in the genome of all bacteria and serve as essential tools for microbial phylogenetic studies and precise species classification (22, 23). Esophageal cancer, due to its complex etiology and large differences in treatment effects, the results of a single study are more limited meta-analysis is a method that can synthesize the results of multiple independent studies, and by aggregating and comparing data from different studies to obtain more accurate and reliable conclusions, it can assess the results of each study as a whole and provide a higher level of evidence support. Meta-analysis study of intestinal flora distribution in esophageal cancer is an important tool to reveal the potential risk factors, prognosis and therapeutic effects of esophageal cancer.

This is a systematic review using meta-analysis of differences in gut microbiome composition between esophageal cancer patients and healthy individuals. The main objective of this study was to summarize and assess the differences in gut microbiome composition and diversity between patients with esophageal cancer and healthy individuals based on data from observational studies, and to systematically explore the association between risk factors and associated factors for esophageal cancer. In addition, we aimed to enable early diagnosis of esophageal cancer and to improve screening and diagnostic efficiency.

## 2 Materials and methods

### 2.1 Data sources and search strategy

This systematic review and meta-analysis follows the PRISMA guidelines for reporting meta-analyses (24). The protocol was prospectively registered at PROSPERO (CRD42023465367). The electronic databases PubMed, Embase, Cochrane Central Register of Controlled Trials, and Web of Science were searched for studies published from the date of each database’s inception up to 25 June 2024 that assessed the changes in the gut microbiota of esophageal carcinoma patients based on 16S rRNA gene sequencing, using the following search terms: (“Esophageal Neoplasms” or “Esophagus Cancers” or “Esophagus” or “Cancer, Esophageal”) and (“16S rRNA” or “Ribosomal RNA, 16S”). The full search strategies used for PubMed are described in **Table 1**.

**Table 1:** Search strategy on PubMed.

#1	“Esophageal Neoplasms”[MeSH]
#2	(((((((((((((((((Esophageal Neoplasms[Title/Abstract]) OR (Esophageal Neoplasm[Title/Abstract])) OR (Neoplasm, Esophageal[Title/Abstract])) OR (Esophagus Neoplasm[Title/Abstract])) OR (Esophagus Neoplasms[Title/Abstract])) OR (Neoplasm, Esophagus[Title/Abstract])) OR (Neoplasms, Esophagus[Title/Abstract])) OR (Neoplasms, Esophageal[Title/Abstract])) OR (Cancer of Esophagus[Title/Abstract])) OR (Cancer of the Esophagus[Title/Abstract])) OR (Esophagus Cancer[Title/Abstract])) OR (Cancer, Esophagus[Title/Abstract])) OR (Cancers, Esophagus[Title/Abstract])) OR (Esophagus Cancers[Title/Abstract])) OR (Esophageal Cancer[Title/Abstract])) OR (Cancer, Esophageal[Title/Abstract])) OR (Cancers, Esophageal[Title/Abstract])) OR (Esophageal Cancers[Title/Abstract])
#3	#1 OR #2
#4	“RNA, Ribosomal, 16S”[MeSH]
#5	((((16S rRNA[Title/Abstract]) OR (rRNA, 16S[Title/Abstract])) OR (16S Ribosomal RNA[Title/Abstract])) OR (RNA, 16S Ribosomal[Title/Abstract])) OR (Ribosomal RNA, 16S[Title/Abstract])
#6	#4 OR #5
#7	#3 AND #6

### 2.2 Outcome measures

To ensure the comparability of the meta-analysis, we standardized the sequencing protocols for all included studies, ensuring that all studies were based on similar variable regions for comparison. Specifically, we selected the V3-V5 region of the 16S rRNA gene because it provides the best resolution and coverage for microbial community analysis. The assessment of microbial diversity can be quantified in terms of the presence of various species within a community (richness) and the equitable distribution of these species (evenness). The collective evaluation of both is commonly referred to as alpha diversity. To assess the alpha diversity of microorganisms, we can use the Shannon and Simpson indices. Also, microbial richness can be assessed by the Chao1 index (i.e., number of species observed/number of operable taxonomic units (OTUs)). These indices provide a quantitative estimate of microbial diversity.

The primary outcomes of interest were: (1) differences in the alpha diversity between esophageal carcinoma patients and healthy control group; (2) differences in gut microbiome composition between esophageal carcinoma patients and healthy control group.

The secondary outcome of interest was to describe microbial taxonomic signatures associated with ESCC.

### 2.3 Inclusion and exclusion criteria

Inclusion criteria were: (1) Patients with a strict diagnosis of esophageal cancer and healthy controls matched for appropriate age, gender, and geographic region. (2) human observational studies comparing the composition of the gut microbiome between patients with esophageal cancer and healthy adults (age ≥18 years), recruited from the general population regardless of race; (3) If studies included a mixed population of children and adults, only those studies presenting their data for adults separately were included; (4) the gut microbiome was measured by means of high-throughput analyses (16S rDNA/rRNA sequencing) in fecal samples. (5) Observational studies, such as cohort or case–control; (6) Outcome indicators including at least one of the following: The diversity or abundance of intestinal flora. studies.

Exclusion criteria were intervention studies and randomized controlled trials. Further exclusion criteria were: (1) cell studies and animal studies; (2) review articles, letters to the editor, case reports, ecological studies, and cross-sectional studies; (3) gut microbiome measured in samples other than feces or by means of culture-dependent techniques or other non-high-throughput sequencing techniques; (4) Studies with incomplete or unreported data; (5) Patients with digestive disorders, such as inflammatory bowel disease and chronic gastrointestinal diseases; (6) Patients who have used drugs with antimicrobial effects or other treatments; (7) Patients with serious concomitant diseases or complications, such as heart disease, liver disease, etc. Studies focusing on specific diseases, written in a language other than English, or published as abstract, editorial or comment were also excluded.

### 2.4 Study selection and data extraction

Two researchers first independently screened the titles of the literature to exclude duplicates, review papers, conference papers, protocols, and communications. The abstracts of the literature were then read by two researchers to identify included and excluded literature. Finally, the remaining literature was read in full by two researchers and further determined for inclusion. Blind double-checking was performed by the researchers during the screening process, and areas of disagreement or uncertainty were discussed and resolved by a third researcher.

The investigators designed and piloted a data extraction form before routine use, and extracted the data independently. For each included study the following information was extracted: study ID (first author and year of publication), country, study design, study population, sample size, method used to measure microbiome, DNA extraction method, platform used, outcomes assessed, results on composition and diversity of gut microbiome in esophageal carcinoma patients versus healthy control group and/or differences in comparison groups, and characterization of microbiome taxonomic signatures in esophageal carcinoma patients.

### 2.5 Risk of bias of individual studies

The Newcastle–Ottawa Scale (25) was used to assess the risk of bias by determining the quality of the observational studies selected using two independent scales (for case–control studies). The scale consists of items divided into three domains: selection, comparison and exposure (case–control studies) or outcome. Studies with a rating of 6 or higher were considered high quality (26). Potential publication bias of each GM abundance was quantitatively assessed by Begg and Egger’s regression intercept tests (27), where a value of p < 0.05 was considered a statistically significant difference for all tests used.

### 2.6 Data analysis

The minimum, mean (M), maximum, and standard deviation (SD) of alpha diversity indexes were extracted. If the median and quartile range in the original data were only provided, we convert it to M and SD. If necessary, Engauge Digitizer was employed to extract digital data from the picture (9). The standardized mean difference (SMD) and 95% confidence interval (CI) of the above indexes between the esophageal carcinoma patients and controls were calculated. Two-sided P-values were statistically significant at less than 0.05. Heterogeneity is represented by I^2^, and 0% means no statistical heterogeneity, I^2^ ≤ 50% adopts a fixed-effects model, and I^2^>50% adopts a random- effects model and analyzes the source of heterogeneity. The results were presented by forest plots and the publication bias by funnel plots. All statistical analyses were conducted using the software STATA, version 15.0 (Stata Corporation, College Station, TX, USA).

## 3 Results

### 3.1 Study and identification and selection

**Figure 1:**
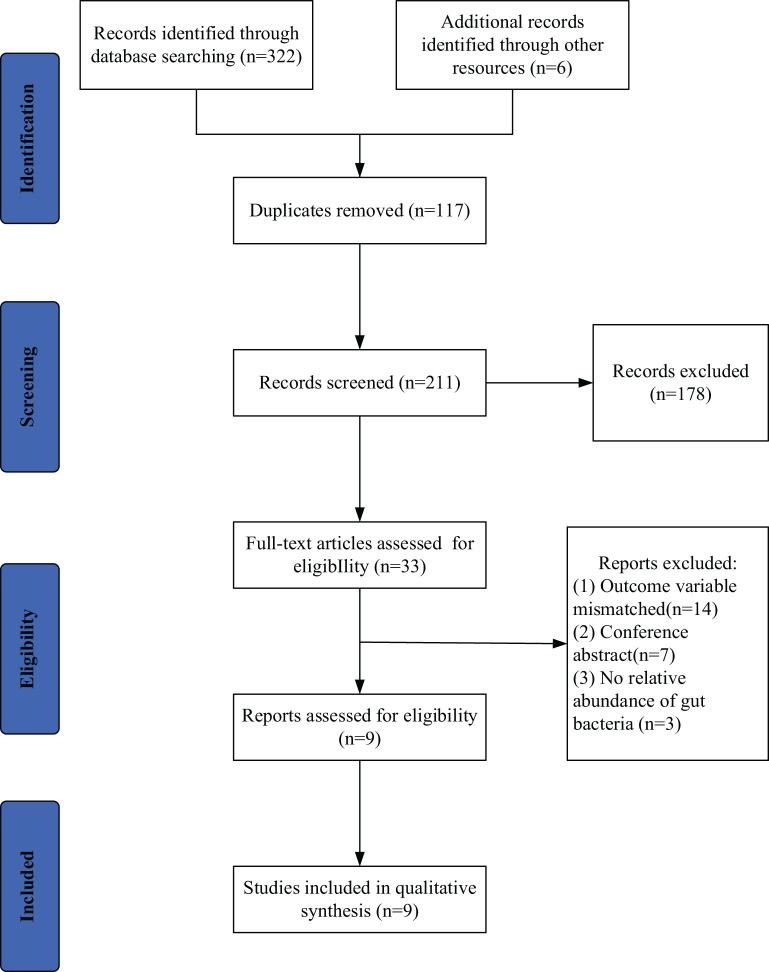
Search and selection procedures of the literature for the systematic review, described in detail by Preferred Reporting Items for Systematic Reviews and Meta-Analyses (PRISMA) flowchart.

A total of 328 studies were retrieved, 117 duplicated studies were removed, 196 studies were removed according to inclusion and exclusion criteria, and 9 studies (9–17) were finally included (PRISMA flowcharts in **Figure 1**). The 9 studies included 216 patients with ESCC and 352 HC. Six (6/9) studies were conducted in China, one (1/9) study was conducted in China and Pakistan, one (1/9) study was conducted in Japan and one was conducted (1/9) in the United States. Details will be shown in **Table 2**.

**Table 2:** Summarizes the clinical and demographic characteristics of the 7 studies.

Author	Country	Year	Population	Age(mean+SD)	Total/male/female	Sample type	Tumor stage	16S region	Sequencing depth	Sequencing Platform
Man Kit Cheung	China	2022	Esophageal Cancer	69 ± 10	15/15/0	Fecal samples	Locally advanced 2 (13.3%), Metastatic 13 (86.7%)	V4	Deep sequencing	Illumina MiSeq
			Health	60 ± 4	16/16/0	Fecal samples				
Ningning Li	China	2022	Esophageal Cancer	NA	40/NA/NA	Fecal samples	locally advanced or stage IV	V4	Deep sequencing	Illumina Hiseq 2500
			Health	NA	147/NA/NA	Fecal samples				
Hirofumi Hasuda	Japan	2023	Esophageal Cancer	69 (55–79)	21/14/7	Fecal samples	NA	V3–V4	Medium depth sequencing	Illumina MiSeq
			Health	51.5 (50–61)	10/8/2	Fecal samples				
YaLi Deng	China	2021	Esophageal Cancer	65.7 ± 4.7	23/4/19	Fecal samples	T1 1(4.3%),T2 3(13.1%),T3 12(52.2), no information7(30.4)	V4	Medium depth sequencing	Illumina MiSeq
			Health	64.3 ± 4.0	23/4/19	Fecal samples				
Hafiz Muhammad Ishaq	China, Pakistan	2021	Esophageal Cancer	53.3 ± 4.4	15/10/5	Fecal samples	NA	V3	Medium depth sequencing	Illumina Hiseq 2500
			Health	NA	10/NA/NA	Fecal samples				
Yuhan Hao	United States	2021	Esophageal Cancer	59.9 ± 9.3	19/18/1	Fecal samples	NA	V3-V5	Deep sequencing	NA
			Health	56.3± 12.8	27/17/10	Fecal samples				
Ningning Li	China	2019	Esophageal Cancer	60(47-72)	13/11/2	Fecal samples	NA	V4	Shallow sequencing	Illumina Hiseq 2500
			Health	55(35-70)	49/28/21	Fecal samples				
Mingjun Gao 2024 (11)	China	2024	Esophageal Cancer	64.2 ± 5.46	20/15/5	Fecal samples	I-II/12, III-IV/8	V4	Medium depth sequencing	Illumina NovaSeq6000
			Health	62.5 ± 4.36	20/15/5	Fecal samples				
Xingqiang Huang 2024 (12)	China	2024	Esophageal Cancer	61.91 ± 5.505	50/34/16	Fecal samples	T1,T2,T3,T4	V3–V4	Medium depth sequencing	Illumina NovaSeq6000
			Health	1.86 ± 6.456	50/34/16	Fecal samples				

### 3.2 Quality of included studies

The included studies were evaluated by Newcastle-Ottawa Scale (9), and six (6/9) studies were ranked with 8*, three (3/9) with 7*, indicating that the quality of the selected studies was generally high. As shown in **Table 3**.

**Table 3:** Quality Assessment of 7 Studies on the Newcastle-Ottawa Scale.

Selection					Exposure				
Study,first author,year	Is the case definition adequate?	Representativeness of the cases	Selection of controls	Definition of controls	Comparability Control for important factor	Ascertainment of exposure	Same method of ascertainment for cases and controls	Non-response rate	Score
Man Kit Cheung 2022 (13)	★	★	★	★	★	★	★	★	8
Ningning Li 2022 (14)	★	★	★	★	★	★	★	★	8
Hirofumi Hasuda 2023 (15)	★	★	★	★	★	★	★		7
YaLi Deng 2021 (10)	★	★	★	★	★	★	★	★	8
Hafiz Muhammad Ishaq 2021 (16)	★		★	★	★	★	★	★	7
Yuhan Hao 2021	★	★	★		★	★	★	★	7
Ningning Li 2019 (17)	★	★	★	★	★	★	★	★	8
Mingjun Gao 2024 (11)	★	★	★	★	★	★	★	★	8
Xingqiang Huang 2024 (12)	★	★	★	★	★	★	★	★	8

*Note: The table above provides a quality assessment of several studies using the Newcastle-Ottawa Scale. Each "★" represents a positive evaluation that a study has met the criteria on a corresponding standard. The final "Score" is the sum of all "★"s, reflecting the overall quality of the study. The higher the score, the better the quality of the study. In the Newcastle-Ottawa Scale (NOS), the maximum score is typically 9 points.

### 3.3 Primary outcomes

#### 3.3.1 Alpha diversity (microbial diversity and richness, microbial dissimilarities)

**Figure 2:**
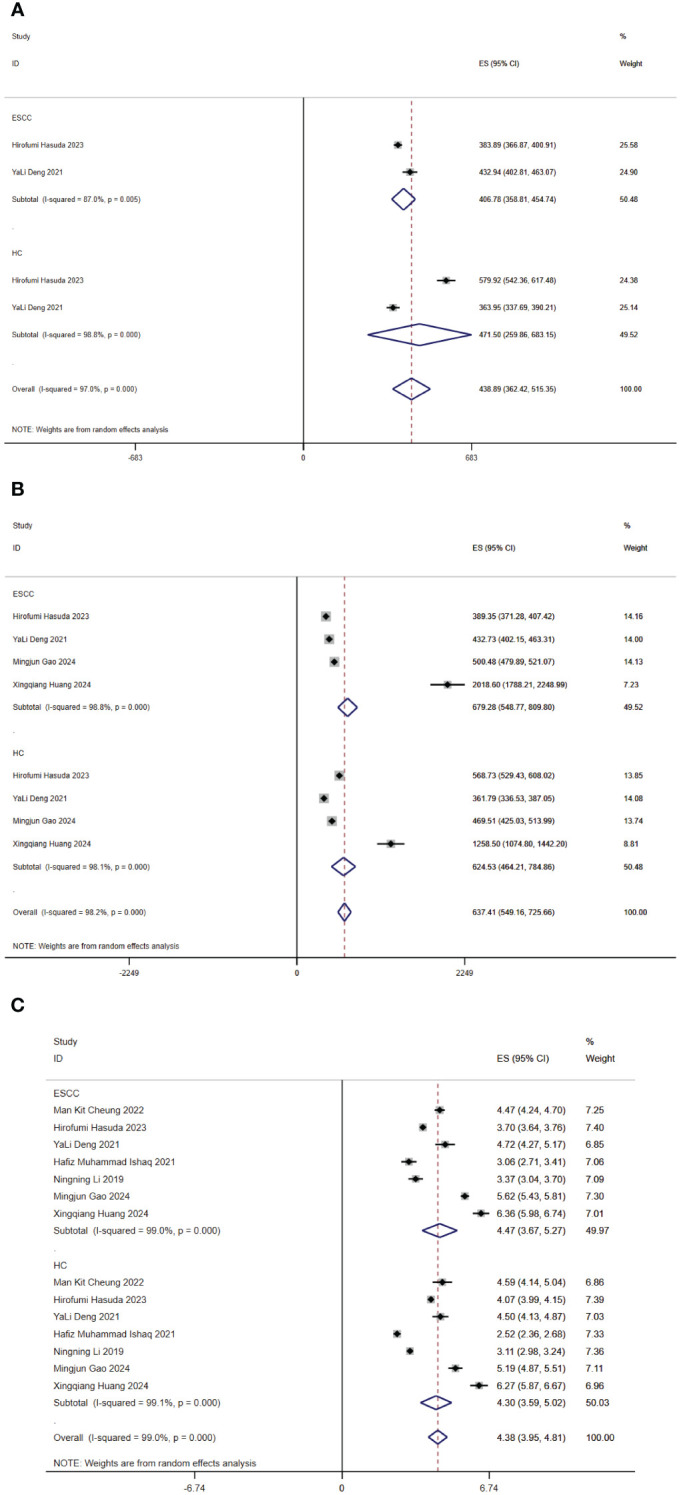
Forest map of alpha diversity differences by ACE **(A)**, Chao1 index **(B)** and Shannon index **(C)**. CI, confidence interval.

Seven (7/9) studies analyzed alpha diversity between ESCC and HC, mainly related to two factors (9): (1) richness, the number of species; (2) diversity, the evenness of individual distribution in a community. The indexes of community richness mainly include Chao1, ACE, Observed species, and Sob. The indexes of community diversity, including Shannon, Simpson, Fisher, and phylogenetic diversity whole tree (PD _whole _tree). A meta-analysis was performed on the alpha diversity indexes reported in two or more studies (**Figure 2**).

Regarding richness, four studies (10–12, 15) provided Chao1 index for quantitative consolidation (ES = 637.41, 95% CI: 549.16 to 725.66, p = 0.000, I^2^ = 98.2%). One study (15) found that the Chao1 index was lower in the esophageal cancer patient group than in the healthy control group. The other studies (10–12) found that the Chao1 index was higher in the esophageal cancer patient group than in the healthy control group. Two studies (10, 15) reported ACE index (ES = 438.89, 95% CI 362.42 to 515.35, p = 0.000, I^2^ = 97%). One study (15) found that the ACE index was lower in the esophageal cancer patient group than in the healthy control group. The other study (10) found that the ACE index was higher in the esophageal cancer patient group than in the healthy control group. In brief, the findings were inconsistent in our included studies.

Regarding diversity, seven studies (10–13, 15–17) reported the Shannon index for quantitative consolidation (ES = 4.38, 95% CI: 3.95 to 4.81, p = 0.000, I^2^ = 99%). The results suggested that two studies (13, 15) the species diversity of gut microbiota decreased in ESCC. Species diversity of the ESCC gut microbiota was elevated in five studies (10–12, 16, 17).

#### 3.3.2 Differences in the microbial composition

Currently, the review identified 9 studies that compared the composition of gut microbiota in patients with ESCC and HC. A meta-analysis was performed on the differentially abundant of gut microbiota reported in two or more studies.

##### 3.3.2.1 At the phylum level

Six (6/9) studies (10–14, 16) described the distinct taxa at the phylum level. Two (2/5) studies found that the relative abundance of Actinobacteria in patients with ESCC was higher than that in HC, and three (3/5) study was lower than in HC (ES = 1.42, 95% CI: 0.28 to 2.55, p = 0.0.185, I^2^ = 28.2%). Three (3/6) studies found that the relative abundance of Bacteroidetes in patients with ESCC was higher than that in HC, and three (3/6) studies was lower than in HC (ES = 37.80, 95% CI: 25.75 to 49.85, p = 0.000, I^2^ = 87.2%). One (1/5) study found that the relative abundance of Firmicutes in patients with ESCC was higher than that in HC, and four (4/5) studies was lower than in HC (ES =42.79, 95% CI: 37.54 to 48.05, p = 0.145, I^2^ = 23.8%). Two (2/5) study found that the relative abundance of Fusobacteria in patients with ESCC was higher than that in HC, and three (3/5) studies was lower than in HC (ES =0.31, 95% CI: -0.36 to 0.87, p = 0.710, I^2^ = 0.0%). Five (5/6) studies found that the relative abundance of Proteobacteria (11, 13, 15, 18, 19) in patients with ESCC was higher than that in HC, and one (1/6) study was lower than in HC (ES =7.48, 95% CI: 5.02 to 9.94, p = 0.04, I^2^ = 2.4%). Four (4/4) studies found that the relative abundance of Verrucomicrobia (10, 11, 13, 14) in patients with ESCC was higher than that in HC (ES =0.09, 95% CI: -0.24 to 0.43, p = 0.726, I^2^ = 0.0%). Two (2/2) studies found that the relative abundance of Tenericutes (10, 14) in patients with ESCC was higher than that in HC. The abundance of Bacteroidetes and Proteobacteria have statistical difference between ESCC and HC. The abundance of Actinobacteria, Firmicutes, Fusobacteria, Verrucomicrobia, and Tenericutes were no statistical difference between ESCC and HC.

##### 3.3.2.2 At the genus level

Six (6/9) studies (9–11, 15–17) described the distinct taxa at the genus level, four (4/4) studies found that the relative abundance of Blautia in patients with ESCC was higher than that in HC (ES =0.31, 95% CI: -0.36 to 0.87, p = 0.710, I^2^ = 0.0%). Two (2/5) studies found that the relative abundance of Bacteroides in patients with ESCC was higher than that in HC, and three (3/5) studies was lower than in HC (ES =0.31, 95% CI: -0.36 to 0.87, p = 0.710, I2 = 0.0%). Two (2/6) studies found that the relative abundance of Faecalibacterium in patients with ESCC was higher than that in HC, and four (4/6) studies was lower than in HC (ES =5.76, 95% CI: 2.92 to 8.60, p = 0.696, I^2^ = 0.0%). Two (2/3) studies found that the relative abundance of Bifidobacterium in patients with ESCC was higher than that in HC, and one (1/3) study was lower than in HC (ES =0.19, 95% CI: -0.52 to 0.90, p = 0.503, I^2^ = 0.0%). Two (2/3) studies found that the relative abundance of Prevotella in patients with ESCC was higher than that in HC, and three (1/3) study was lower than in HC (ES =0.58, 95% CI: -0.77 to 1.92, p = 0.382, I^2^ = 5.4%). One (1/2) study found that the relative abundance of Alistipes (ES =2.47, 95% CI: -1.12 to 6.06, p = 0.872, I^2^ = 0.0%), Subdoligranulum (ES =1.55, 95% CI: -1.20 to 4.30, p = 0.854, I^2^ = 0.0%), Dialister (ES =1.86, 95% CI: -1.26 to 4.97, p = 0.788, I^2^ = 0.0%) in patients with ESCC was higher than that in HC, and one (1/2) study was lower than in HC. Three (3/3) studies found that the relative abundance of Megamonas (ES =2.27, 95% CI: -1.03 to 5.57, p = 0.456, I^2^ = 0.0%) in patients with ESCC was lower. Three (3/3) studies found that the relative abundance of Clostridium sensu stricto 1 (ES =2.49, 95% CI: -0.32 to 5.30, p = 0.933, I^2^ = 0.0%) in patients with ESCC was higher. Two (2/2) studies found that the relative abundance of Fusicatenibacter (ES =2.13, 95% CI: -1.09 to 5.35, p = 0.940, I^2^ = 0.0%), Anaerostipes (ES =1.36, 95% CI: -1.22 to 3.93, p = 0.459, I^2^ = 0.0%), Klebsiella (ES =0.83, 95% CI: -1.57 to 3.22, p = 0.111, I^2^ = 50.2%), Clostridium (ES =1.53, 95% CI: -0.96 to 4.02, p = 0.817, I^2^ = 0.0%), and Ruminococcus (ES =0.36, 95% CI: -0.85 to 1.58, p = 0.604, I^2^ = 0.0%) in patients with ESCC was lower. Two (2/2) studies found that the relative abundance of Lachnoclostridium (ES =3.77, 95% CI: -0.47 to 8.02, p = 0.947, I^2^ = 0.0%), Veillonella (ES =0.80, 95% CI: -1.18 to 2.79, p = 0.509, I^2^ = 0.0%), Lactobacillus (ES =0.89, 95% CI: -1.18 to 2.96, p = 0.236, I^2^ = 0.0%), Enterococcus (ES =0.10, 95% CI: -0.60 to 0.80, p = 0.629, I^2^ = 0.0%) in patients with ESCC was higher. Two (2/4) studies found that the relative abundance of Streptococcus (ES =0.01, 95% CI: -0.13 to 0.15, p = 0.667, I^2^ = 0.0%) (11, 13, 15, 18, 19) in patients with ESCC was higher than that in HC, one (1/4) study was lower than in HC. and one (1/4) study found no difference between ESCC and HC. One (1/3) study found that the relative abundance of Dorea (ES =0.01, 95% CI: -0.16 to 0.18, p = 0.681, I^2^ = 0.0%) in patients with ESCC was higher than that in HC, and two (2/3) studies was lower than in HC. Three (3/3) studies found that the relative abundance of Roseburia (ES =1.64, 95% CI: -0.36 to 3.64, p = 0.648, I^2^ = 0.0%) in patients with ESCC was lower. There was no significant difference between ESCC and HC in the abundance of genera.

##### 3.3.2.3 At the family level

Two (2/7) studies (13, 16) described the distinct taxa at the family level. Two (2/2) studies found that the relative abundance of Bacteroidaceae (ES =30.96, 95% CI: 19.03 to 42.89, p = 0.381, I^2^ = 0.0%), Enterobacteriaceae (ES =3.93, 95% CI: -1.14 to 9.00, p = 0.493, I^2^ = 0.0%) and Rikenellaceae (ES =3.91, 95% CI: -1.14 to 8.97, p = 0.591, I^2^ = 0.0%) in patients with ESCC was higher than that in HC. One (1/2) study found that the relative abundance of Lachnospiraceae (ES =9.03, 95% CI: 1.53 to 16.54, p = 0.855, I^2^ = 0.0%) and Ruminococcaceae (ES =13.93, 95% CI: 4.89 to 22.97, p = 0.935, I^2^ = 0.0%) in patients with ESCC was higher than that in HC, and one (1/2) study was lower than in HC.

### 3.4 Publication bias

**Figure 3:**
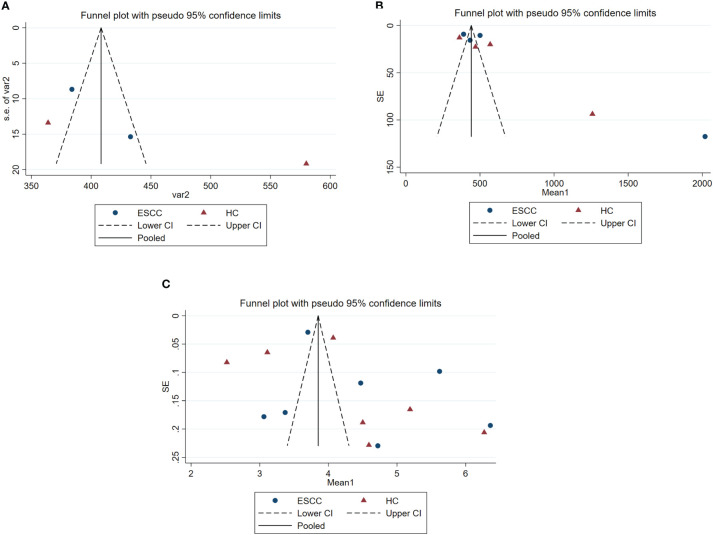
Funnel plots of gut microbiota analysis results, estimating potential publication bias of the included studies for each study. SE, standard error. **(A)** represents the funnel plot for assessing publication bias in ACE studies. **(B)** represents the funnel plot for assessing publication bias in Chao 1 studies. **(C)** represents the funnel plot for assessing publication bias in Shannon’s studies.

The Begg and Egger’s regression intercept tests confirmed that ACE and Shannon’s were not significantly biased by publication bias. The Chao1 results have significant publication bias. As shown in **Table 4**. We constructed separate funnel plots for all outcome indicators to test for possible publication bias. Visual inspection of the funnel plots did not reveal any significant publication bias. Details as shown in **Figure 3**.

**Table 4:** P value for Egger’s and Begg’s tests for publication bias.

	P for Egger’s test	P for Begg’s test
ACE	0.252	0.157
Chao1	0.017	0.013
Shannon’s	0.176	0.352

## 4 Discussion

ESCC patients often lack obvious symptoms in the early stages, leading to poor prognosis. Endoscopic biopsy or swab sampling is an invasive procedure that hinders routine screening for early-stage ESCC. Whether the detection of fecal microbiota can explain the microbial characteristics of patients with upper gastrointestinal tumors is a question worth discussing. In recent years, more studies have found that the pathogenesis and development of non-intestinal tumors is also affected by the intestinal microbiome (28, 29). Thus, fecal microbiota may be significant for further investigation of the features of esophageal cancer. In the present systematic review, we compared the distribution of the gut flora of ESCC and HC, and the study found differences in the composition of the gut microbiome between the two. A total of 7 studies were included, including 146 patients with ESCC and 282 HC. Regarding richness, two (2/3) studies found that the Chao1 index and ACE index were lower in the esophageal cancer patient group than in the healthy control group. In a significant proportion of studies (4/6), the species diversity of the ESCC gut microbiota Shannon’s diversity has increased. By contrast, a significant difference was observed in the alpha diversity of these studies.

The composition of the gut microbiota is significantly different in patients with esophageal cancer (EC) compared to healthy individuals, with an increased richness of gut microbiota observed in EC patients. At the phylum level, multiple studies have consistently found that the relative abundance of Actinobacteria, Proteobacteria, Verrucomicrobia, and Tenericutes is higher in patients with ESCC compared to healthy controls. Conversely, more studies have found that the relative abundance of Firmicutes and Fusobacteria is lower in ESCC patients. Bacteroidetes has shown varying results, with some studies reporting higher relative abundance in ESCC patients compared to healthy controls. Previous studies on colorectal cancer have also found an increase in Bacteroidetes and a decrease in Firmicutes compared to healthy individuals (30). The complex microbial environment in the human gut is mainly composed of bacteria, with Firmicutes and Bacteroidetes being the most abundant phyla, followed by Actinobacteria, Proteobacteria, and Fusobacteria. Normally, Bacteroidetes and Firmicutes are the predominant phyla in the gut of healthy adults, while Actinobacteria are relatively scarce (31). Changes in the abundance of Firmicutes, Actinobacteria, and Bacteroidetes, have been associated with intestinal flora disorder and inflammation in EC patients. These findings suggest a potential association between the gut microbiota profile and tumors in the upper digestive tract, indicating the involvement of certain beneficial bacterial taxa in the development of esophageal, similar to what has been observed in colorectal cancer.

At the genus level, more studies have found that the relative abundance of Actinobacteria, Bifidobacterium, Lachnoclostridium, Veillonella, Lactobacillus, and Enterococcus is higher in patients with esophageal squamous cell carcinoma (ESCC) compared to healthy controls (HC). Conversely, more studies have found that the relative abundance of Faecalibacterium, Fusicatenibacter, Anaerostipes, Klebsiella, Clostridium, Ruminococcus, Dorea, Roseburia, and Megamonas is lower in ESCC patients. Four studies have reported a higher relative abundance of Bacteroides in ESCC patients compared to HC. Previous studies using quantitative PCR have shown that Bifidobacterium and Lactobacillus are significantly reduced in the gut microbiota of esophageal cancer patients (32). Megamonas has also been found to be reduced in the gut microbiota of patients with multiple system atrophy, which may be related to intestinal inflammation (18). It has been reported that Alistipes promotes the development of right-sided colon tumors through the IL-6/STAT3 pathway, but some studies suggest that Alistipes may have beneficial effects in immunotherapy (19). The abundance of Alistipes in the fecal microbiota is positively correlated with the production of tumor necrosis factor TNF, indicating that the decrease in Alistipes may be associated with reduced effectiveness of immunotherapy (33). Streptococcus and Lactobacillus genera are lactic acid producers, and the accumulation of lactic acid plays a crucial role in carcinogenesis, including angiogenesis, cell migration, and metastasis (10). Streptococcus has also been identified as a potential diagnostic biomarker for EC. Additionally, the combined abundance of Streptococcus and Prevotella in tumor tissues is a potential prognostic biomarker for ESCC (34). Streptococcus may have promising implications in the diagnosis and/or prognosis of EC and ESCC (35).

At the family level, two (2/2) studies found a higher relative abundance of Bacteroidaceae, Enterobacteriaceae, and Rikenellaceae in patients with ESCC compared to healthy controls. This is consistent with previous research on individuals with type-II diabetes and their gut microbiota (36). On the other hand, Veillonellaceae and Prevotellaceae were found to be significantly lower in esophageal cancer patients compared to healthy volunteers, which aligns with current research (37, 38). It has been reported that the Lachnospiraceae NK4A136 group is positively correlated with enhanced intestinal barrier function in mice, and the abundance of Agathobacter is decreased in EC patients.Veillonellaceae has been associated with beneficial effects, such as the production of T-regulatory immune cells (39). These alterations in gut bacteria may have an impact on host health, even though the progression of the disease may not be directly related to the intestine (40).

The uniqueness of this review lies in its comprehensive multidimensional analysis approach, which not only assesses the richness and diversity of the gut microbiota but also delves into the composition of the microbiota at the phylum, genus, and family levels. This meticulous analytical strategy transcends the limitations of a single indicator, providing a more comprehensive and in-depth perspective on the complex changes in the gut microbiota in esophageal cancer. Additionally, this discussion particularly emphasizes the heterogeneity of the research findings and deeply explores the significant differences in the results between different studies through the quantification of heterogeneity (I^2^ statistic). Such differences may stem from various factors, such as study design, sample processing, and sequencing techniques. Attention to and analysis of heterogeneity help readers understand why the changes in the gut microbiota in esophageal cancer are so complex and variable. In summary, this review, through its unique analytical methods and in-depth discussion of heterogeneity, provides valuable insights into the field of esophageal cancer gut microbiome research and lays the foundation for further research and clinical applications.

## 5 Conclusion

In conclusions, we observed differences in the composition of the gut microbiome between ESCC and HC patients in our studies at the portal, genus and family levels. Alterations in specific flora or combinations of flora may be associated with the development and progression of esophageal cancer. Studying the distribution of intestinal flora in patients with esophageal cancer can help to further understand the mechanism of the relationship between intestinal flora and esophageal cancer, reveal its association with esophageal cancer, and provide scientific evidence for the prevention, diagnosis, and treatment of esophageal cancer.

The findings of this study underscore the potential role of the gut microbiome in the etiology of esophageal cancer, providing a novel perspective for further exploration of the relationship between gut microbiota and the disease. These studies are expected to provide new ideas and methods for early diagnosis, treatment and prevention of esophageal cancer. While this study offers valuable insights, it is not without limitations. To overcome these constraints, we recommend that future research should adopt standardized methodologies, expand sample sizes, and utilize longitudinal study designs to enhance the reliability and generalizability of the findings. Moreover, the integration of multi-omics data analysis will contribute to a more comprehensive understanding of the complex interplay between the gut microbiota and esophageal cancer. In the future, larger cohort studies are needed to further investigate the differences in the gut microbiome in the ESCC spectrum.

## Data Availability

The original contributions presented in the study are included in the article/supplementary material. Further inquiries can be directed to the corresponding author.
